# The Great Debate at “Melanoma Bridge”, Naples, December 7th, 2019

**DOI:** 10.1186/s12967-020-02340-w

**Published:** 2020-04-16

**Authors:** Paolo A. Ascierto, Sanjiv S. Agarwala, Alexander Eggermont, Jeffrey E. Gershenwald, Jean-Jacques Grob, Omid Hamid, Olivier Michielin, Michael Postow, Igor Puzanov, Hassane M. Zarour, Corrado Caracò, Alessandro Testori

**Affiliations:** 1 Unit of Melanoma, Cancer Immunotherapy and Innovative Therapy, Istituto Nazionale Tumori IRCCS “Fondazione G. Pascale”, Via Mariano Semmola, 80131 Naples, Italy; 2 Temple University School of Medicine, Philadelphia, PA USA; 3 Princess Máxima Center Research Directorate, CS 3584 CS Utrecht, The Netherlands; 4 Department of Surgical Oncology and Cancer Biology, The University of Texas MD Anderson Cancer Center, Houston, TX USA; 5 Hopital de la Timone, Aix-Marseille University, Marseille, France; 6 Angeles Clinic & Research Institute, Santa Monica, CA USA; 7 Oncology Service, Precision Oncology Center, Oncology Department, Centre Hospitalier Universitaire Vaudois (CHUV), Lausanne, Switzerland; 8 Memorial Sloan Kettering Cancer Center and Weill Cornell Medical College, New York, NY USA; 9 Roswell Park Comprehensive Cancer Center, Buffalo, NY USA; 10 Hillman Cancer Center, University of Pittsburgh, Pittsburgh, PA USA; 11 Department Melanoma, Soft Tissue, Muscle-Skeletal and Head-Neck, Istituto Nazionale Tumori IRCCS “Fondazione G. Pascale”, Naples, Italy; 12 Dermatology, Fondazione IRCCS Policlinico San Matteo, Pavia, Italy

**Keywords:** Melanoma, Staging immunotherapy, Anti-PD-1, Anti-CTLA-4, Targeted therapy, BRAF inhibitor, MEK inhibitor, Adjuvant, Neoadjuvant

## Abstract

The Great Debate session at the 2019 Melanoma Bridge congress (December 5-7, Naples, Italy) featured counterpoint views from experts on five topical issues in melanoma. These were whether to choose local intratumoral treatment or systemic treatment, whether patients with stage IIIA melanoma require adjuvant therapy or not, whether treatment is better changed at disease progression or during stable disease, whether adoptive cell transfer (ACT) therapy is more appropriate used before or in combination with checkpoint inhibition therapy, and whether treatment can be stopped while the patient is still on response. As was the case for previous meetings, the debates were assigned by meeting Chairs. As such, positions taken by each of the melanoma experts during the debates may not have reflected their respective personal approach.

## Introduction

The Great Debate session at the 2019 Melanoma Bridge congress (December 5-7, Naples, Italy) featured counterpoint views from experts on five topical issues in melanoma. These were whether to choose local intratumoral treatment or systemic treatment, whether patients with stage IIIA melanoma require adjuvant therapy or not, whether treatment is better changed at disease progression or during stable disease, whether adoptive cell transfer (ACT) therapy is more appropriate used before or in combination with checkpoint inhibition therapy, and whether treatment can be stopped while the patient is still on response. As was the case for previous meetings, the debates were assigned by meeting Chairs. As such, positions taken by each of the melanoma experts during the debates may not have reflected their respective personal approach.

## Local or systemic treatment?

### Sanjiv S. Agarwala: in favor of local treatment

Intralesional oncolytic therapy (ILOT) is the direct injection of tumors with agents that can result in local tumor regression and that may have a systemic effect that is also immunologically mediated [1]. ILOT can be viral-based (e.g. talimogene laherparepvec [T-VEC], HF-10, coxsackievirus A21 [CVA21, CAVATAK™]), or non-viral based (e.g. PV-10, interleukin [IL]-12). These agents can offer a high local concentration with palliation and symptom control and can also induce host immune anti-tumor activity, augmenting the local response as well as providing a durable response in distant and non-injected regional metastases with limited systemic toxicity.

Clinical trials of T-VEC, CVA21 and PV-10 have demonstrated clinically significant, durable responses. In the phase III OPTiM trial, the primary end point of durable response rate (objective response lasting continuously ≥ 6 months) was significantly higher with T-VEC compared with GM-CSF ((16.3% [95% CI 12.1–20.5] versus 2.1% [95% CI 0–4.5]; odds ratio [OR], 8.9; P < 0.001) in 436 patients with unresected stage IIIB-IV melanoma and regional metastases [4, 5]. Overall response rate (ORR) was also higher in patients treated with T-VEC (26.4% versus 5.7%), with 41% of patients in the T-VEC group having a complete response (CR). Interim overall survival (OS) analysis also suggested a benefit with T-VEC, with median OS of 23.3 months versus 18.9 months with GM-CSF. This benefit was most pronounced in patients with stage IIIB/C, IVM1a disease. A second oncolytic virus is the naturally occurring common cold ICAM-1-targeted RNA virus, CVA21. In the phase II CALM study of 57 patients with stage IIIC-IV melanoma, the primary end point of investigator-assessed immune-related progression-free survival (PFS) at 6 months was 38.6% [4]. Median OS was 26 months and 1-year survival rate was 75.4%. ORR was 28.1% and responses were observed in injected lesions, non-injected non-visceral lesions and in distant non-injected visceral lesions.

PV-10 is a sterile, nonpyrogenic 10% solution of rose bengal disodium, a small molecule fluorescein derivative. In a phase II trial, ORR by-patient was 71% (CI 51–87%) with 50% CR (CI 31–69%) in the subgroup of 28 patients who received PV-10 into all existing melanoma lesions (i.e. no non-injected lesions) [3].

Intralesional therapy may also offer potential synergy when combined with systemic therapy. In particular, immune stimulation effected by intralesional agents may promote the release and presentation of tumor-derived antigens which may synergise with the systemic effects of checkpoint inhibitor agents. Several ongoing clinical trials are assessing the potential of combining oncolytic immunotherapy with checkpoint inhibitors. In a phase II trial of T-VEC in combination with ipilimumab versus ipilimumab alone, the combination resulted in a significantly higher ORR (39% versus 18%; OR, 2.9 [95% CI 1.5–5.5; P = 0.002) in 198 patients with unresectable stages IIIB-IV melanoma [8]. At three-year follow-up, ORR remained significantly higher with the combination (36.7% versus 16.0%; OR, 3.0; 95% CI 1.6–6.0; P = 0.002) [9]. Median PFS was 13.5 months with the combination and 4.5 months with ipilimumab alone and median OS was not reached in either arm. Total and activated CD8 T cells in the peripheral blood have been shown to increase after T-VEC administration and further increase after combined T-VEC and ipilimumab [37]. In a phase 1b/2 study of PV-10 in combination with pembrolizumab in patients with advanced cutaneous melanoma, safety and tolerability was acceptable with a CR rate for injected lesions of 77% (3% partial response [PR]) achieved after a median of three injections/lesion [2].

Further potential for the intralesional approach is as neoadjuvant therapy in surgically resectable disease. In a phase II, randomized, open-label trial with T-VEC in 150 patients with resectable stage IIIB, IIIC or IVM1a melanoma, the addition of neoadjuvant T-VEC improved two-year recurrence-free survival (RFS) (hazard ratio [HR] 0.75, P = 0.07) and OS (HR 0.49, P = 0.050) compared with surgery alone [10].

In favor of ILOT, monotherapy has been shown to be effective in locoregional disease while combination with immunotherapy has a synergistic effect without overlapping toxicity and may reverse tumor resistance.

### Olivier Michielin: in favor of systemic treatment

Although intratumoral therapy may be considered a new paradigm for cancer immunotherapy, it actually dates back to the late 19th century and ‘Coley’s toxins’ for the treatment of malignant tumors. However, there has been an increased focus on this area in the past decade with T-VEC therapy now approved and an integral part of our therapeutic options in inoperable stage III-IV melanoma. Despite this, more needs to be understood and prospectively evaluated in order to make the best use of T-VEC and other intratumoral compounds in development.

In the phase II trial of T-VEC in combination with ipilimumab, although the primary endpoint of ORR did reach statistical significance with the combination versus ipilimumab alone, the secondary endpoints of PFS and OS were not significantly improved [9].

A successful intratumoral approach has several key requirements. These include the availability of accessible and injectable lesions, the potential for high local concentrations to be achieved with the delivery of adequate injection volume and the ability to reside in the tumor bed for sufficient time to produce a biological effect without diffusion out of the lesion. In addition, intratumoral agents should be able to overcome local resistance mechanisms, and have the ability to reprogram the tumor microenvironment (TME) and engage an abscopal effect.

**Fig. 1:**
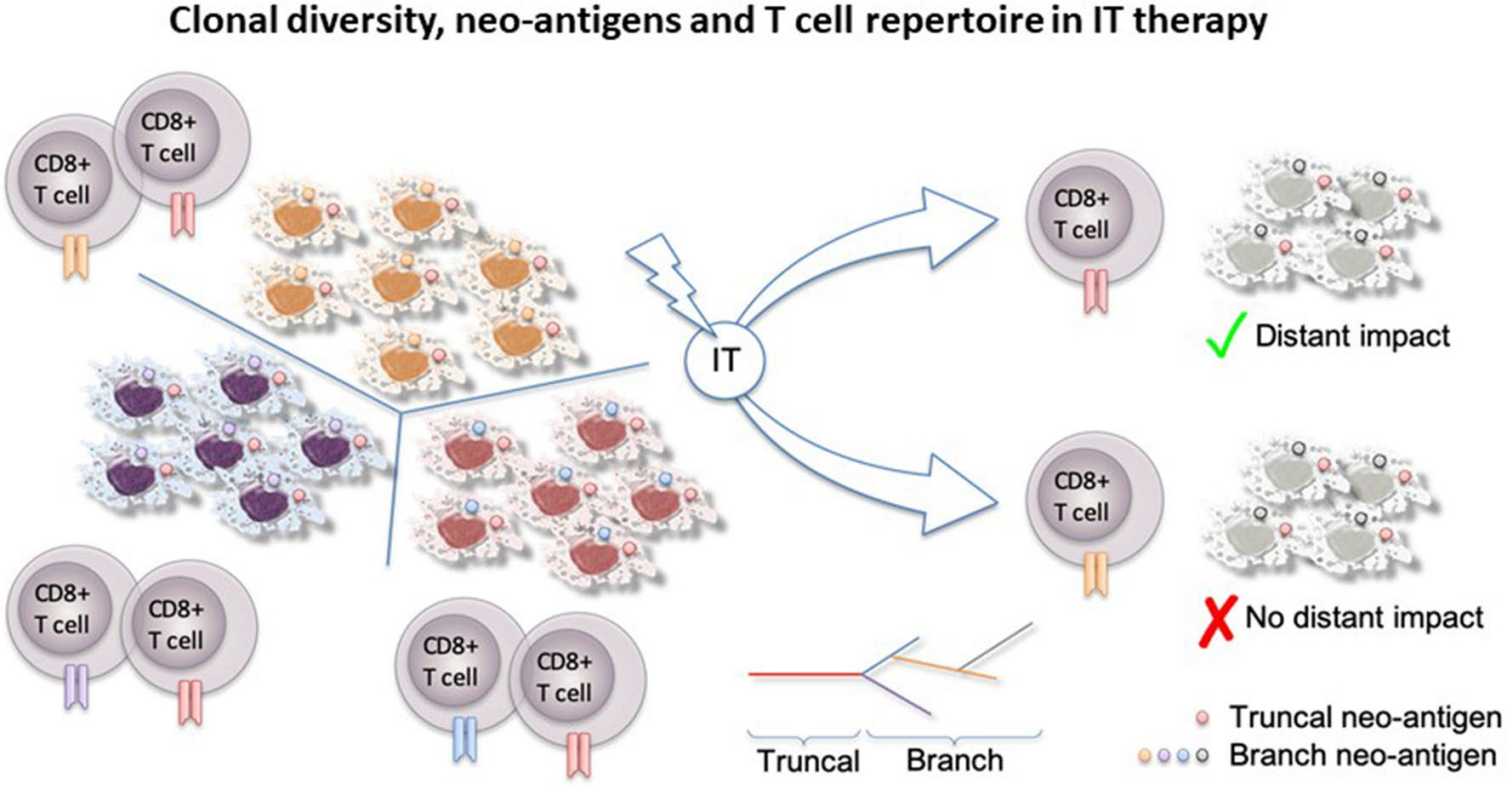
Clonal diversity, neo-antigens and T cell repertoire in IT therapy

An important consideration and potential limitation of intratumoral therapy is the differential biology at the injection site and distant sites. Intratumoral injection must overcome immune local escape mechanisms but biology may be very different from that of the non-injected lesion. Repertoire and activation status of T cells may vary and may or may not have a distant impact Fig. 1. T cell receptor repertoire heterogeneity within the tumor or between tumor sites was demonstrated within the TRACERx project, which showed that expanded T cell receptors can be subdivided into ubiquitous, i.e. those found in all tumor regions, and regional, i.e. those present in a subset of tumor regions [26]. The number of ubiquitous and regional T cell receptors correlates with the number of ubiquitous and regional non-synonymous mutations, respectively. T cells elicited by intratumoral therapy and specific for regional mutations might not confer effective systemic immunity for the patient.

Another issue is that accessibility of lesions may very likely create a strong bias. Injection into subcutaneous and lymph node tumors may influence T cell repertoire and T cell homing capacity. Indeed, this process has been shown to be tightly pre-programmed and there are concerns over intratumoral injections typically limited to superficial lesions.

The pharmacokinetics (PK) and pharmacodynamics of intratumoral injection may also represent a limitation of this approach. Local PK data can be very different from whole-body kinetics. For example, intratumoral injection of nivolumab involves diffusion to a near infinite dilution, with PK driven by off-rate and diffusion. Serum half-life for systemic nivolumab is 28 days but molecular dissociation half-life is around 2.7 h, meaning the effective intratumoral concentration might be short-lived. However, it should be noted that such limitations are not present with intratumoral viruses.

A final consideration is where best to integrate intratumoral therapies in the current treatment landscape. It may have an important role alone or in combination with checkpoint blockade in neoadjuvant therapy, helping maximize the pathological CR rate and increasing the T cell repertoire. It may also be useful as a treatment for local relapse after adjuvant treatment. In patients with stage IV disease, intratumoral treatment may be an option when standard immunotherapy alone fails. However, tumor heterogeneity may be a limitation in this setting.

In conclusion, intratumor therapies have interesting properties, yet important aspects need to be addressed, including tumor heterogeneity and T cell repertoire, injection site bias and intratumoral PK/PD. More translational studies are needed to efficiently guide their clinical development and identify their optimal place in the rapidly evolving melanoma landscape.

## Key points

**Fig. 2:**
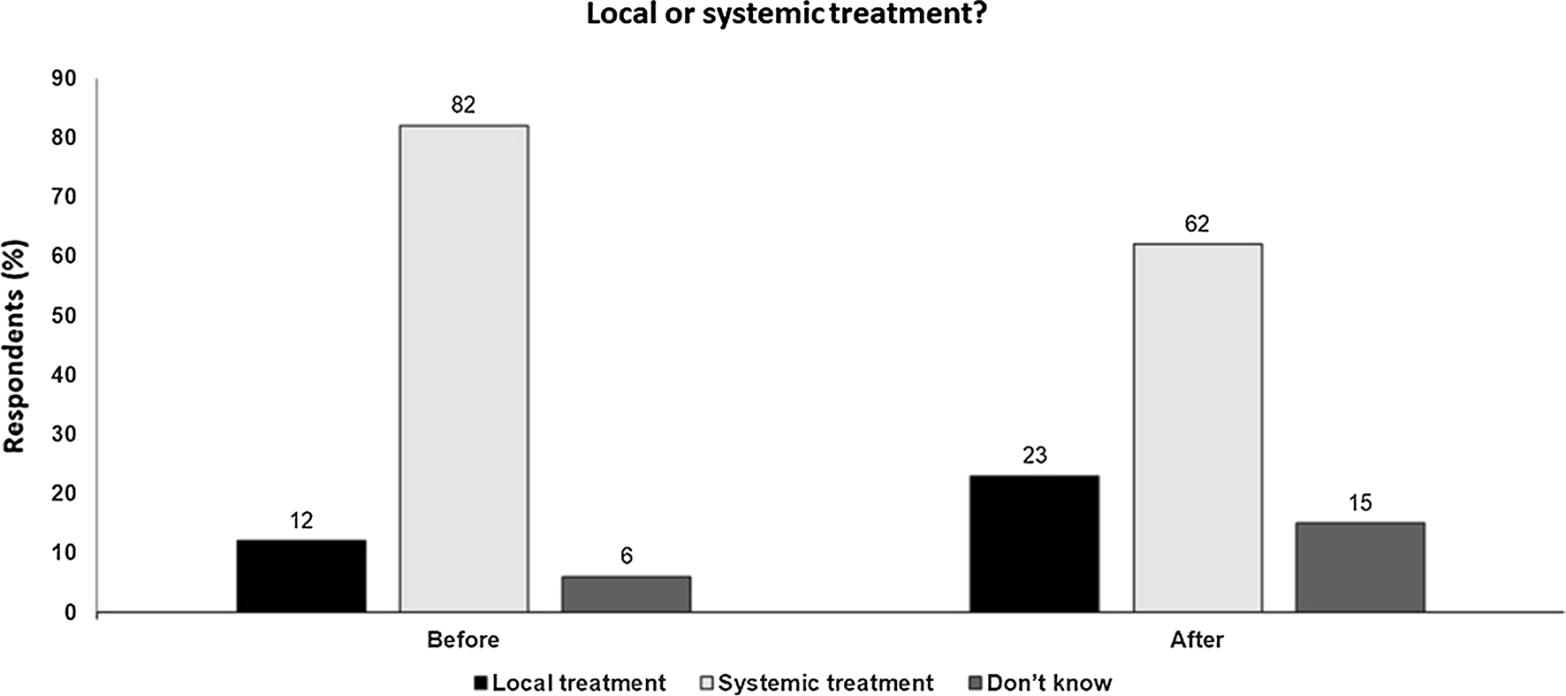
Local or systemic treatment Audience response before and after debate

- Clinical trials of ILOT (T-VEC, CVA21 and PV-10) have demonstrated clinically significant, durable responses.
- These agents induce host immune anti-tumor activity, augmenting the local response as well as providing a response in distant and non-injected regional metastases with limited systemic toxicity.
- The success of ILOT requires accessible and injectable lesions, the potential for high local concentrations, the delivery of adequate injection volume and the ability to reside in the tumor bed for sufficient time.
- In addition, intratumoral agents should be able to overcome local resistance mechanisms, and have the ability to reprogram the TME and engage an abscopal effect.
- Immune stimulation effected by intralesional agents may synergise with the systemic effects of checkpoint inhibitor agents and clinical trials have indicated a synergistic effect that may reverse tumor resistance and without overlapping toxicity.
- More studies are needed to efficiently guide the clinical development of ILOT and identify its optimal place in the rapidly evolving melanoma landscape (Fig. 2).

## Do patients with stage IIIA disease really need adjuvant therapy?

### Alexander Eggermont: Yes

The current situation with checkpoint inhibitors and targeted treatments in the adjuvant setting directly parallels that previously seen in the advanced disease setting. The first adjuvant trial was with ipilimumab, which showed an absolute difference in survival of 11% versus placebo at both one and 5 years [12]. OS at 5years was 65% in the ipilimumab group compared with 54% in the placebo group (HR for death, 0.72; 95.1% CI 0.58–0.88; P = 0.001). This benefit was sustained long-term, with an 8.7% absolute difference at 7 years for OS [13]. Targeted therapy with dabrafenib plus trametinib is also effective for patients with BRAF V600 mutant melanoma [25], with an estimated three-year recurrence-free survival (RFS) rate of 58% versus 39% with placebo (HR for relapse or death, 0.47; 95% CI 0.39–0.58; P < 0.001). However, unlike with ipilimumab, the survival benefit declines after the 1st year and is not sustained longer-term. From about 2years, immunotherapy offers a greater survival benefit than targeted treatment.

However, ipilimumab is not acceptable as adjuvant therapy because of the high rate of immune-related adverse events. Consideration of unacceptable side effects is more important in the adjuvant setting given that, depending on the risk of relapse and efficacy of the treatment, a subset of patients will not gain any benefit from treatment. In the adjuvant ipilimumab trial, many of the immune-related adverse events were grade 1–2 thyroiditis which did not require intervention, but some were serious and chronic, including nephritis, colitis and insulin-dependent diabetes. Overall, 53% of patients discontinued because of toxicity.

Given the unacceptable toxicity of ipilimumab, the anti-PD-1 agents nivolumab and pembrolizumab represent more appropriate adjuvant treatment options. In the CheckMate-238 trial, the 1-year rate of RFS was 70.5% (95% CI 66.1–74.5) with nivolumab versus 60.8% (95% CI 56.0–65.2) with ipilimumab (HR for disease recurrence or death, 0.65; 97.56% CI 0.51–0.83; P < 0.001) [50]. Treatment-related grade 3–4 adverse events were reported in 14% of the patients in the nivolumab group and in 46% of patients in the ipilimumab group. Similarly, pembrolizumab significantly increased one-year rate of RFS versus placebo (HR for recurrence or death, 0.57; 98.4% CI 0.43–0.74; P < 0.001) in a phase III trial in 1019 patients [11]. Treatment-related grade 3–5 adverse events were reported in 14.7% of the patients in the pembrolizumab group.

In this trial, the 15-month incidence of immune-related adverse events was 37% with pembrolizumab versus 9% with placebo [15]. The incidence of adverse events with pembrolizumab was 29% at 6months, so reducing treatment duration to 6 months would not have a major impact on reducing adverse event rate or improving the risk: benefit ratio of treatment. However, the occurrence of an immune-related adverse event was significantly associated with a longer RFS in the pembrolizumab arm (HR 0.61), but not the placebo arm. Thus immune-related adverse events may be an indicator of a beneficial host immune response [15].

It is also important to consider the effect of adjuvant treatment in patients at different substages of stage III disease. Based on the American Joint Committee on Cancer (AJCC) 7th edition melanoma staging system [6], pembrolizumab prolonged RFS versus placebo in patients with stage IIIA melanoma (HR 0.32) as well as patients with stage IIIB (HR 0.57) and IIIC (HR 0.58) disease [14]. After reclassification of patients using the AJCC 8th edition melanoma staging system [19], the RFS benefit of pembrolizumab was similarly observed across AJCC-8 stage III disease subgroups [14]. Based on these results, patients with stage IIIA disease, especially younger patients, should be considered for adjuvant treatment.

In patients with stage I-II disease, it is necessary to identify those patients at high-risk of relapse. More than 50% of deaths due to melanoma occur in stage I/IIA sentinel lymph node biopsy (SLNB) negative patients, generally considered to be low risk, so there is a need to identify high-risk SLNB-negative patients. The Mayo Clinic CP-GEP model, currently in development for possible clinical use, combines clinical pathologic variables with a gene expression profiling algorithm of metastasis predisposition genes and has been shown to be of potential prognostic benefit in identifying SLNB negative patients at risk of relapse [33, 34]. Use of a profiler may ultimately be informative for clinical decision-making.

The discussion whether all stage IIIA patients should be recommended adjuvant therapy is of course a risk:benefit ratio discussion. With relatively rare but potentially chronic immune-related adverse event, in a relatively low risk situation (especially in the AJCC-8 stage IIIA) the situation is clearly different for a patient aged over 70 years compared to a 25-year-old. The recommendations are clearly described in the most recently published guidelines [16, 17].

### Jeffrey E. Gershenwald: No

In patients with AJCC 8th edition stage IIIA melanoma, adjuvant therapy may reduce the risk of recurrence and improve survival following initial treatment and in whom there is no evidence of disease. However, adjuvant therapy can only provide a benefit if melanoma persists and can be of no benefit to patients with no risk of recurrent disease. Thus, a fraction of patients who receive adjuvant therapy have no potential benefit, but are still at risk of toxicity. Although relatively infrequent, adverse effects related to treatment can result in chronic life-long sequelae, which may be of particular concern in younger patients.

Implementation of the AJCC 8th edition melanoma staging system [19] that includes revisions to the definition and number of stage III stage groups, has impact on patient counseling and management, as well as contemporary adjuvant clinical trial design and assessment. Completion lymph node dissection (CLND) is no longer routinely offered as initial management for SLN-positive patients; nonetheless, it is important to note that accurate nodal staging is still important in order to identify patients at risk of relapse. There is an at least theoretical concern that since CLND is no longer routinely performed for the vast majority of SLNB-positive patients, some potentially clinically useful data, such as upstaging stage IIIA patients given that non-sentinel node involvement is an independent predictor of poor survival, may impact on clinical decision-making. However, patients with AJCC 8th edition stage IIIA disease are overall unlikely to harbour tumor-involved non-SLNs if a CLND were to be performed, and so are very unlikely to be upstaged.

In the CheckMate-238 trial of nivolumab versus ipilimumab, no patients with AJCC 7th edition stage IIIA disease were included [Weber 2017], while the EORTC 1325/KEYNOTE-54 trial of pembrolizumab included AJCC 7th edition stage IIIA patients but only those with > 1 mm metastasis in the sentinel node [11]. In the subgroup of patients with AJCC 7th edition stage IIIA disease, RFS at one-year was 89.8% with pembrolizumab and 76.8% with placebo (HR 0.32, 99% CI 0.09–1.23; P = 0.0217) [13]. When the trial was stratified according to the new AJCC 8th edition melanoma staging system, disease stage was prognostic but not predictive of response [13], which is not unexpected given that these pathological features have not previously been associated with response to anti-PD-1 therapy. Note that the AJCC 8th edition-staged patients from the trial are still relatively enriched for “higher risk” patients given the requirement that if they were AJCC 7th edition IIIA, the SLN metastasis had to have a tumor burden > 1 mm. In patients with AJCC 8th edition stage IIIB, a clear RFS benefit was observed. However, in patients with AJCC 8th edition stage IIIA disease, although there appears to be a possible signal of RFS benefit, confidence intervals are wide and follow-up is thus far limited. As such, this should be considered very preliminary data for generally low-risk patients. Given that toxicity is not stage-specific, balancing the risk of serious toxicity versus potential benefits becomes an increasingly important consideration in this patient group.

The COMBI-AD study had similar eligibility criteria for stage IIIA patients, i.e., SLN metastasis > 1 mm [28]. RFS benefit was observed across all AJCC 8th edition stage III subgroups, but the benefit was less clear in stage IIIA disease [22]. It also needs to be recognised that as the melanoma community considers treating earlier stage disease in the adjuvant setting, short-term follow-up can be deceptive and a note of caution is required. It is important to emphasize that there was a variable and selective approach to adjuvant trial design, so that even within stage groups, patients were higher risk and represent enriched risk populations rather than a typical distribution.

The use of individualized risk models incorporating multiple features is becoming increasingly important. It is likely that we are entering an era in which decisions regarding adjuvant therapy will be based on these types of risk models that incorporate a multitude of clinicopathological and ultimately molecular and immune factors. There are already multiple high-quality biomarkers that can be integrated into risk models (e.g. primary tumor mitotic rate, SLN tumor burden, among others). Conventional staging will likely continue to inform decision-making but will not be a sole criterion.

To summarize, clinical benefit is only possible if residual subclinical disease exists. Toxicity may occur in any patient who receives adjuvant therapy, with the potential for life-long sequalae. The role of adjuvant therapy for patients with ‘low-risk’ stage IIIA disease continues to evolve and it may be better to reduce toxicity risk by minimizing potential overtreatment through the use of validated clinical tools and models. An overriding question also remains—do we need to treat in the adjuvant setting, or can patients be treated only if and when melanoma ultimately recurs. Finally, the high financial cost of these treatments, both to the individual and at a health care provider level, is a very real concern.

## Key points

**Fig. 3:**
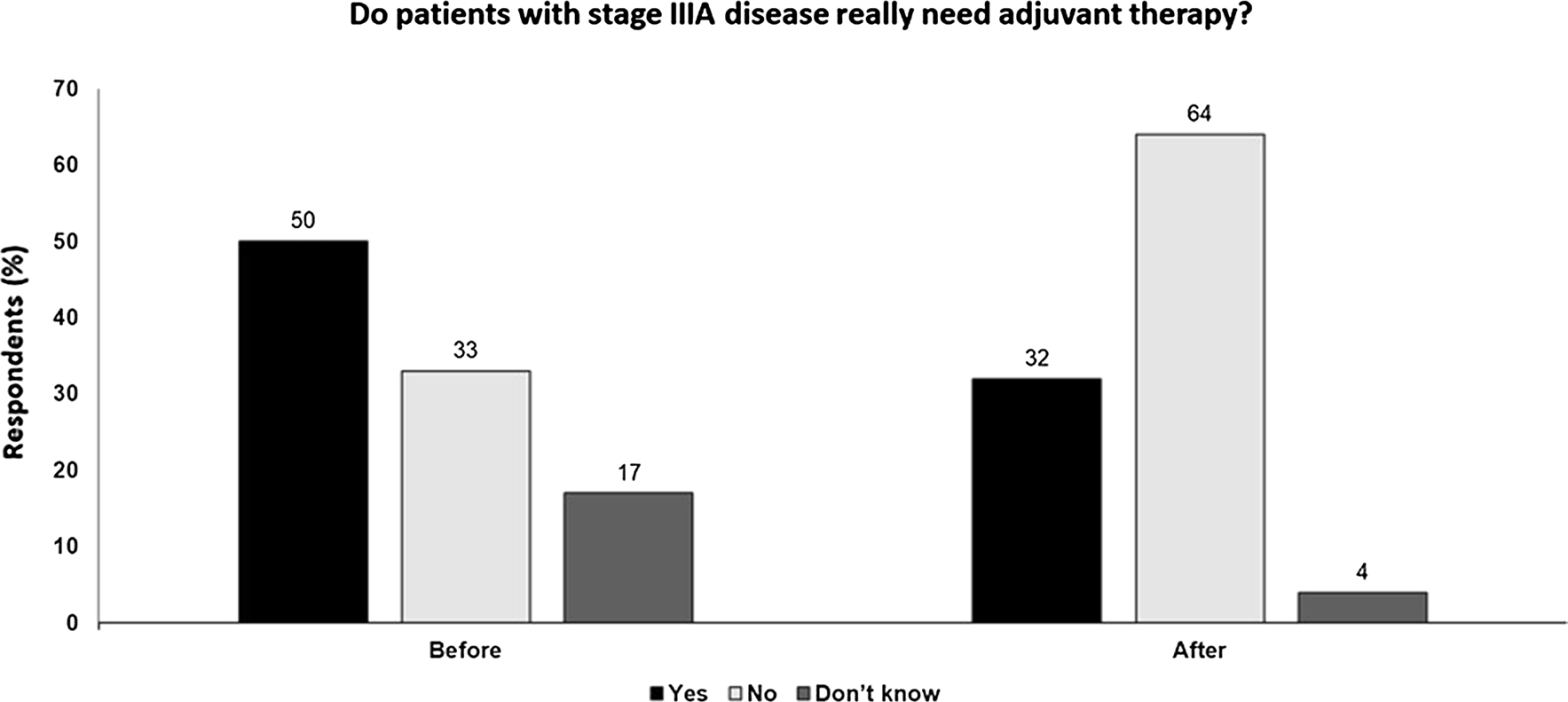
Do patients with stage IIIA disease really need adjuvant therapy? Audience response before and after debate

- Several trials have now shown a benefit of checkpoint inhibitors as adjuvant therapy, with anti-PD-1 inhibition more acceptable due to lower toxicity than ipilimumab.
- Targeted therapy with dabrafenib plus trametinib is also effective, but the survival benefit is not sustained longer-term and immunotherapy offers a greater survival benefit from about 2 years.
- The benefit of PD-1 blockade is observed across AJCC-8th edition stage III disease subgroups and patients with stage IIIA disease, especially younger patients, should be considered for adjuvant treatment.
- However, adjuvant therapy can only provide a benefit if melanoma persists and is of no benefit to patients with no risk of recurrent disease. Balancing the risk of serious toxicity versus potential benefit is especially important in stage IIIA patients.
- Although infrequent, adverse effects related to treatment can result in chronic life-long sequelae, which may be a particular concern in younger patients.
- Decisions regarding adjuvant therapy will increasingly be based on risk models that incorporate a multitude of clinicopathological as well as molecular and immune factors (Fig. 3).

## When is the best time to change treatment on anti-PD1 therapy: at disease progression or during stable disease?

### Michael Postow: in favor of switching at disease progression and not stable disease

One argument for switching systemic therapy at disease progression rather than during stable disease (SD) is that patients with SD can have prolonged SD. Looking at patients treated with nivolumab, many patients with SD, which included some with significant tumor shrinkage, remain stable for a sustained period and did not ultimately progress [48]. Moreover, an analysis of pooled data from the KEYNOTE-001 and 006 trials of pembrolizumab showed similar OS outcomes at 5 years whether patients had a partial response (PR) or SD at week 24 [20]. As would be expected, complete responders at week 24 did better than patients with PR/SD. Given that treatment for responders would not be switched, there does not seem a valid rationale for switching patients with SD after 24 weeks from start of therapy.

Patients with SD should also not switch based upon their week 12 assessment. Although overall survival outcomes for patient with SD at week 12 do look worse than patients with PR or CR at week 12, there are patients with SD at week 12 who become responders by week 24, and, as mentioned, above, if patients with SD at week 12 remain SD at week 24, their long term OS appears similar to patients with PR. Overall, these data in aggregate are insufficient to recommend patients with SD at week 12 should switch therapy.

In addition, no multivariable analysis has been performed on the apparent association between tumor response and overall survival. In fact, response was shown to be related to known prognostic factors, including baseline tumor size, ECOG performance status and metastatic stage. Therefore, the apparent finding that SD patients have lower OS than patients with PR/CR may be confounded by worse prognostic features.

Furthermore, second-line therapy after PD1 progression can still be effective. There is no clinical evidence that second-line therapy is less effective if used to treat patients after disease progression rather than patents with prior SD on anti-PD1. Ipilimumab has been shown to be just as effective after anti-PD-1 therapy as before, with response rates of 10–15% [51], and response rates to anti-PD-1 s are similar whether before or after ipilimumab [21]. Anti-PD-1 s also show similar outcomes when used after progression on ipilimumab with or without BRAF inhibitors [27]. Moreover, switching from anti-PD-1 s to BRAF/MEK inhibitors can be associated with significant toxicity problems involving treatment interruption or dose reductions [40]. Thus, switching patients with SD has the potential of causing toxicity issues before such a change in systemic therapy is truly needed.

To conclude, SD on anti-PD-1 therapy should not be considered a terrible event. The apparent inferior OS outcomes could all be due to the simple fact that patients with SD have a worse prognosis at baseline compared to patients who have a CR or PR. Patients with SD 24 weeks into anti-PD1 agents have the same overall survival as responders, and since we do not know who these patients are at week 12, patients with SD should not be switch to other therapy at week 12 as well. Second-line therapies can still be effective after disease progression and there are no data that suggest treatment intensification or switch at SD improves outcomes compared to changing therapy at PD.

### Omid Hamid: in favor of switching during stable disease

Although it may be true that the majority of patients with CR are those with a low tumor burden, these patients typically progress to high tumor burden and M1C disease. We also do not have many treatment options post-PD-1 progression and it is not possible to return patients to the same stage they were previously at after the disease has progressed.

In the analysis of data from the pooled KEYNOTE-001 and 006 trials of pembrolizumab, patients with SD at week 12 but no response or progression at weeks 18 or 24 are more relevant to this argument than those with SD at week 12 and a subsequent response at week 18 or 24. In these patients, OS rates decline rapidly from2 years with a survival rate of around 50% after 5 years. Similarly, with nivolumab plus ipilimumab, OS in patients with stable disease is only 50% at 2 years [29] and there is a higher rate of CR and PR in patients alive at 5 years. In patients treated with dabrafenib plus trametinib, OS at 2 years for those with SD as best response was only 29% [35]. These are not great outcomes and it is important to understand why these patients are not doing better.

In a retrospective analysis of patients with advanced BRAF-mutant melanoma, switching from targeted therapy to checkpoint inhibitors during ongoing response resulted in an OS benefit compared with switching at time of disease progression [38]. However, no significant correlation was seen between time of switching and PFS or response rates to checkpoint inhibitor therapy.

There are more accurate means to assess how patients with SD are actually responding to treatment. Various imaging modalities may help predict outcomes. In a retrospective analysis of patients treated with anti-PD-1-based immunotherapy, [18F]2-fluoro-2-deoxy-d-glucose positron emission tomography (FDG-PET) imaging better predicted long-term outcomes compared with standard computed tomography (CT) response criteria [46]. Non-invasive PET imaging of T cells can be used for cancer diagnostics, disease monitoring, and patient stratification. PET Response Criteria in Solid Tumors (PERCIST) 1.0 have been shown to be more sensitive and accurate than RECIST 1.1 for the detection of an early therapeutic response to chemotherapy in patients with non-small-cell lung cancer (NSCLC) [43]. In melanoma, complete metabolic response in the first BRAF/MEK inhibition treatment cycle predicted OS in patients with advanced BRAF-mutant disease [32]. Circulating tumour DNA (ctDNA) analysis may also predict relapse following resection in stage II-III melanoma [47] and studies have reported that ctDNA detection is associated with clinical outcomes across cancer types. These assessments should be included with current management paradigms; early evaluation with biopsy and ctDNA analysis supported by imaging can help in treatment switching decision-making. Further randomized trials will also help reveal the optimal time for switching and best overall treatment strategy.

## Key points

**Fig. 4:**
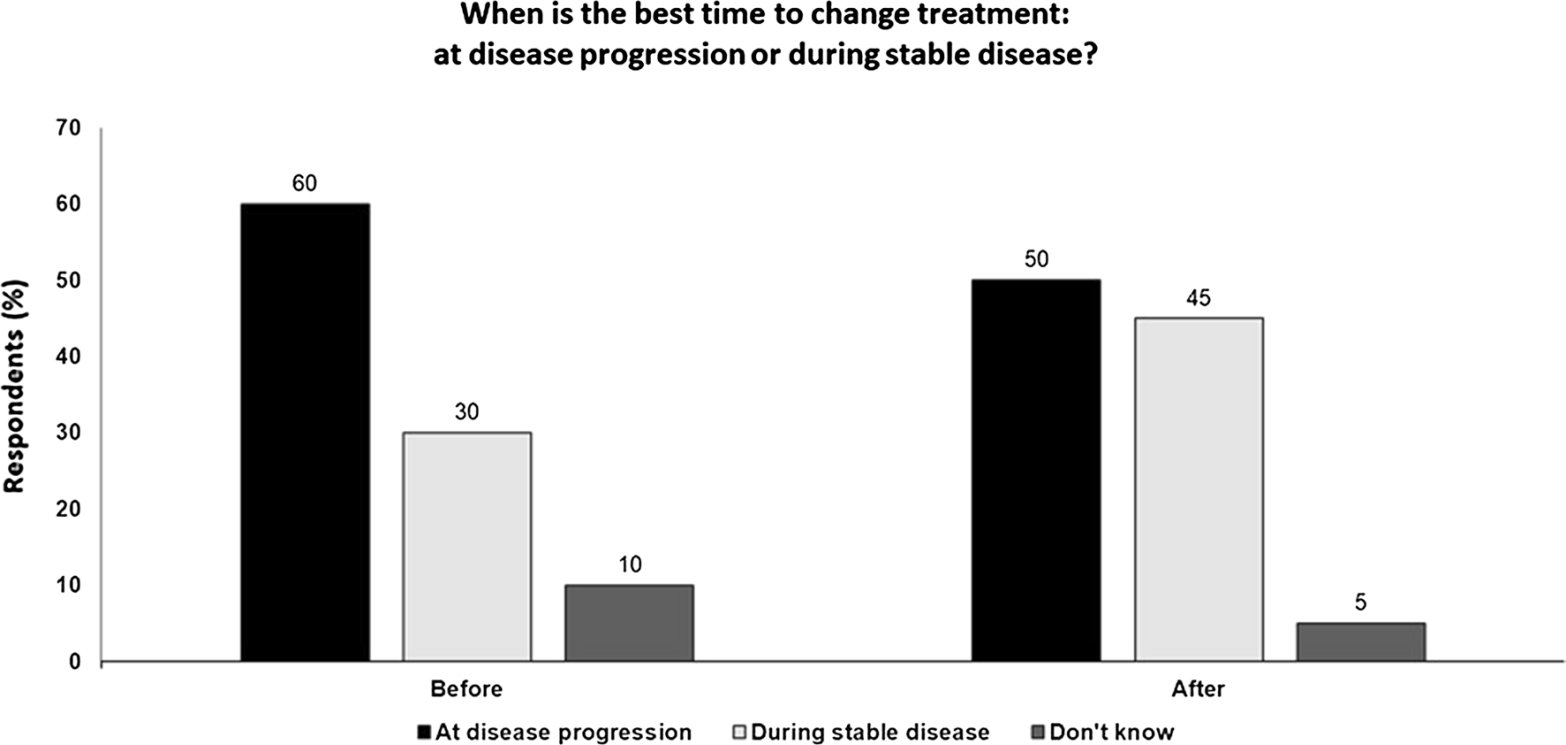
When is the best time to change treatment on anti-PD1 therapy: at disease progression or during stable disease? Audience response before and after debate

- SD may be considered a positive response and a reflection of poor prognostic features and may not itself may not be independently associated with a poor outcome.
- Patients with SD may remain stable for a sustained period and not ultimately progress and long-term OS outcomes may be irrespective of response at week 24.
- Second-line therapy can be as effective if used to treat patients after disease progression rather than patents with SD.
- Moreover, switching therapy (e.g. from anti-PD-1 s to BRAF/MEK inhibitors) can be associated with significant toxicity problems.
- However, there are limited treatment options post-PD-1 progression and it is not possible to return patients to the same stage they were previously after progression.
- Other approaches to assess how patients with SD are actually responding may be more useful for treatment switching decision-making, e.g. ctDNA analysis and imaging (Fig.  4).

## Adoptive cell transfer before or in combination with checkpoint inhibitors

### Hassan M. Zarour: in favor of before

Evidence for ACT utilizing tumor-infiltrating lymphocytes (TILs) in melanoma is encouraging with multiple clinical trials reporting response rates of approximately 40–50% with 10–20% CRs. These responses have been shown to be durable and have been observed in both treatment-naïve and refractory patients.

C-144-01 is a phase II trial of ACT using lifileucel (LN-144) in patients with stage IIIc-IV metastatic melanoma who have received at least one prior treatment with systemic therapy including an immune checkpoint inhibitor. In 42 primary refractory patients enrolled in the trial, with a best response of progressive disease (PD) on their first anti-PD-1/L1 treatment, an ORR of 41% was observed [41]. Responses were durable with median duration of response not reached after one year of follow-up.

In an earlier trial, 80 patients with stage IV melanoma were enrolled, of which 57 were treated with ACT and 23 were withdrawn mainly due to clinical deterioration during TIL preparation i.e. 28% drop-off [7]. Although ORR was 40% for treated patients, this was reduced to 29% for all enrolled patients. This trial also provided evidence that anti-CTLA-4 therapy after ACT provides clinical benefit, with three of 19 (16%) patients who received ipilimumab after TIL therapy experiencing ongoing CRs.

ACT of TILs has many challenges. It is very expensive and requires technical expertise. There are also issues over the time required to generate TILs; this is typically 4–8 weeks during which time some patients may progress. In addition, the rate of success in generating TILs may vary, although is usually > 75% so is not generally a limiting factor. Most of the data are from small studies and non-randomized trials and a prospective randomized study is required.

The optimal clinical setting for ACT in melanoma is also unclear. Treatment-naïve patients have many potent treatment options, including checkpoint blockade, so this does not appear to be an appropriate setting. A more logical place is in anti-PD-1 and targeted treatment-refractory patients. However, the question of whether PD-1 therapy before ACT would improve its efficacy is currently unknown, with no clear evidence in the literature. In fact, it has also recently been reported that PD-1 blockade in sub-primed CD8 cells induces anti-PD-1 therapy resistance through the induction of dysfunctional PD-1^+^CD38^hi^ cells [49]. In inflamed tumors, ACT may not be needed since various therapies can be used to overcome resistance mechanisms. However, ACT may be important when T-cells cannot reach the tumor i.e. in immune-excluded or immune-desert phenotypes. In these settings, the rationale for combined therapy to improve ACT goes beyond immune checkpoint blockade and additional strategies to allow T cell infiltration into the tumor may be required.

### Igor Puzanov: in favor of combination

CD19-engineered CAR-T cells are clinically active, especially in liquid tumors, although not all patients achieve durable responses and toxicity (e.g. cytokine-release syndrome, neurotoxicity) may be an issue. In solid tumors, CAR-T cells need to successfully traffic from the blood into solid tumor sites, despite potential T cell chemokine receptor-/tumor-derived chemokine mismatches, as well as infiltrate the stromal elements of tumors in order to elicit tumor-associated antigen (TAA)-specific cytotoxicity, regardless of antigen loss or heterogeneity. Even after successful trafficking and infiltration, T cells become rapidly dysfunctional owing to a hostile TME, that can include oxidative stress, hypoxia and the presence of inhibitory soluble factors and cytokines, suppressive immune cells, Tregs, and myeloid derived suppressor cells (MDSCs), and T cell-intrinsic negative regulatory mechanisms, e.g. checkpoint inhibitory receptors. CAR-T cells themselves also have the potential for immunogenicity and toxicity.

Various strategies may be adopted to help overcome a hostile TME. CAR-Ts that deplete fibroblast cells, or that degrade the extracellular matrix can overcome physical and metabolic barriers. CAR-Ts that interrupt inhibitory adenosine and PGE2 signaling, and CAR-Ts expressing dominant negative TGFβ can be used to prevent inhibition by tumor-derived soluble factors and cytokines. The presence of immunosuppressive immune cells may be addressed by the simultaneous depletion of MDSCs and Tregs e.g. through the use of alternative homeostatic cytokines, such as IL-7 and IL-21, to boost CAR-T efficacy without stimulating Tregs. Intrinsic regulatory mechanisms of T cells may be overcome by combining CAR-T therapy and PD-1 blockade, the use of PD-1 switch receptors to neutralize inhibitory PD-1 signaling, blocking CTLA-4 enhanced adoptive transfer, or engineering CAR-T cells lacking inhibitory molecules (e.g., diacylglycerol kinase).

Generating CAR-T cells can take 3–4 weeks but many patients expect immediate treatments. Because of this, anti-PD-1 may be given to bridge the gap before the CAR-T cells are available. However, PD-1 blockade prior to antigen priming with cancer vaccine can abrogate the anti-tumor immune effect [49]. In a murine model, HPV16 E7 tumor vaccine or anti-PD-1 alone had little effect on tumor volume while the combination of vaccine and anti-PD-1 significantly reduced tumor burden. However, starting anti-PD-1 therapy before the vaccine abrogated the beneficial effect of the combination. A similar effect was seen with regard to survival. Although this scenario involved using a vaccine in a murine model, this may have clinical relevance to how ACT and immunotherapy may interact in patients.

In addition, simultaneous addition of anti-PD-1 negated the antitumor effects of the vaccine with anti-OX40 antibody in mice [44]. Vaccine plus anti-OX-40 produced a tumor response. However, when anti-PD-1 was added, antigen-specific CD8 + T-cell tumor infiltration, antitumor response and survival were all reduced, indicating that anti-PD-1 added at the start of therapy exhibits a detrimental effect. Addition of anti-PD-1 later i.e. end of treatment sequence, was not detrimental but was not associated with any additional improvement in tumor volume.

In practice, many patients eligible for ACT will have previously received anti-PD-1 therapy. Given this, the use of anti-PD-1 s after ACT may not be of major clinical utility. CAR-T therapy may have significant toxicity, both on-target off-tissue cross-reactions when a targeted tumor antigen is also expressed on other tissues and off-target cross-reactions that occur when the engineered receptor cross-reacts with an unanticipated stereochemically-related antigen that is present on an essential tissue. The financial toxicity of CAR-T may also be a problem for many patients. To conclude, anti-PD-1/PD-L1 should be an automatic backbone for checkpoint inhibitor-based therapy, and combinations with other modalities, including CAR-T and other cell therapies needs to be developed in a rational manner.

## Key points

**Fig. 5:**
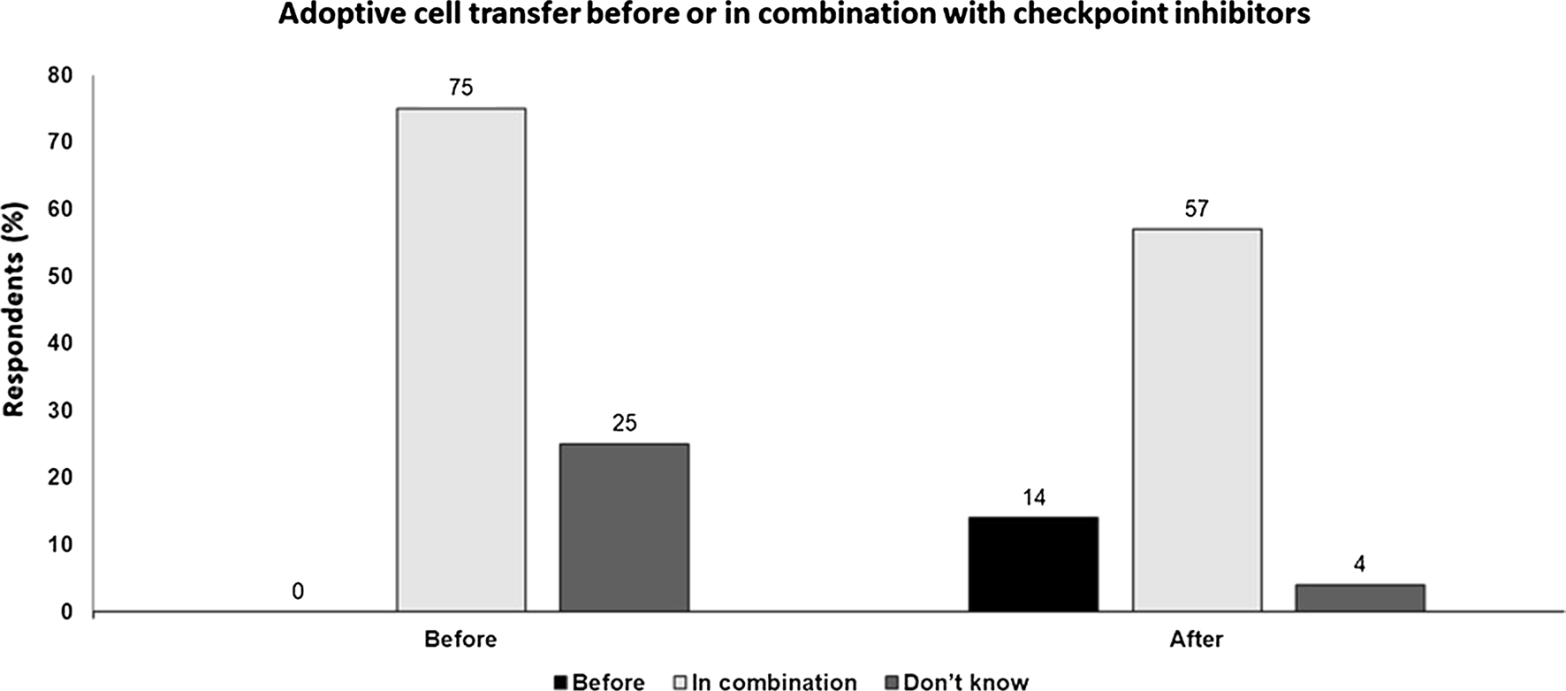
Adoptive cell transfer before or in combination with checkpoint inhibitors Audience response before and after debate

- Evidence for ACT utilizing TILs in melanoma is encouraging with multiple clinical trials reporting response rates of approximately 40–50% in both treatment-naïve and refractory patients.
- Treatment-naïve melanoma patients have many treatment options, including checkpoint blockade, so this does not appear to be an appropriate setting and so use in anti-PD-1 and targeted treatment-refractory patients may be more logical.
- Anti-PD-1 may be given to bridge the gap before the CAR-T cells are available. However, PD-1 blockade prior to antigen priming with cancer vaccine can abrogate the anti-tumor immune effect in mice, and may have clinical relevance to how ACT and immunotherapy are combined.
- In practice, many patients eligible for ACT will have previously received anti-PD-1 therapy so the use of anti-PD-1 s after ACT may not be of major clinical utility.
- ACT may be important when T-cells cannot reach the tumor i.e. in immune-excluded or immune-desert phenotypes.
- Anti-PD-1/PD-L1 should be the backbone of therapy, and combinations with other modalities including ACT need to be developed in a rational manner (Fig. 5).

## Can we stop treatment while on response?

### Jean-Jacques Grob: Yes

Even a short duration of immunotherapy can modulate the TME and activate an active immune reaction which may be durable. Moreover, there is concern that over-activation of the immune system via long-term inhibition of PD-1/PD-L1 may be deleterious by leading to immune exhaustion. thereby allowing tumor growth as well as compromising self-tolerance and inducing chronic auto-immune disease. There are also societal concerns regarding the sustainability, financial cost and burden on the healthcare system of chronic long-term immunotherapy. Given all these factors, stopping checkpoint blockade while patients are on response may be potentially beneficial. However, the evidence for such an approach is limited.

Data from the adjuvant setting suggests stopping treatment while on response may be possible, since the difference in RFS between PD-1 treatment and control arms in adjuvant trials is accounted for by those patients who respond, discontinue on response after 1year and are still responding [11]. Although there is no randomized trial in metastatic melanoma, discontinuation of nivolumab after 1year in patients with metastatic NSCLC resulted in worse PFS compared with continuous nivolumab treatment [45]. However, the PFS plateau of PFS in the one-year treatment arm shows that discontinuation did not compromise benefit in a subset of patients. Retrospective analyses of metastatic melanoma trials, and real-world data also suggest that benefit can be maintained after stopping treatment. In exploratory analysis of the KEYNOTE-006 trial, 86% of patients who completed 2 years on pembrolizumab were progression-free at 20 months after end of therapy [30]. In clinical practice, a study across 14 centers in Europe and Australia reported disease progression in only 40 of 185 (22%) patients who discontinued pembrolizumab or nivolumab in the absence of disease progression or treatment-limiting toxicity [24].

There are also criteria which can help select the best patients for safe discontinuation. Intensity of response to anti-PD-1 treatment is a prognostic marker and prolonged PFS can be expected in patients with a CR or PR who do not progress before 2–3 years [24, 30]. Discontinuation after CR has been shown to be safe; among complete responders, only 4 of 67 (6.0%) who discontinued pembrolizumab for reasons other than progression had disease progression in the KEYNOTE-001 trial [39]. The 24-month disease-free survival (DFS) rate from time of CR in the 67 patients was 90%. In patients treated with nivolumab plus ipilimumab, severe toxicity may be indicative of who will have a durable benefit. In trials of nivolumab plus ipilimumab, patients who discontinued for toxicity in the induction phase had comparable PFS and OS to those who did not discontinue due to treatment-related adverse events [23, 42].

Finally, stopping while on response is an option because there is a chance for rescue via rechallenge with anti-PD-1 s, surgery/stereotactic radiosurgery or BRAF/MEK inhibitor treatment in patients who relapse after discontinuation.

However, even in the best situation there is always a risk. Some relapses may be immediately life-threatening (e.g. brain metastasis) and the efficacy of rechallenge, or a new therapy line is never guaranteed. The acceptance of this risk by the patient is crucial in any decision to stop treatment.

**Fig. 6:**
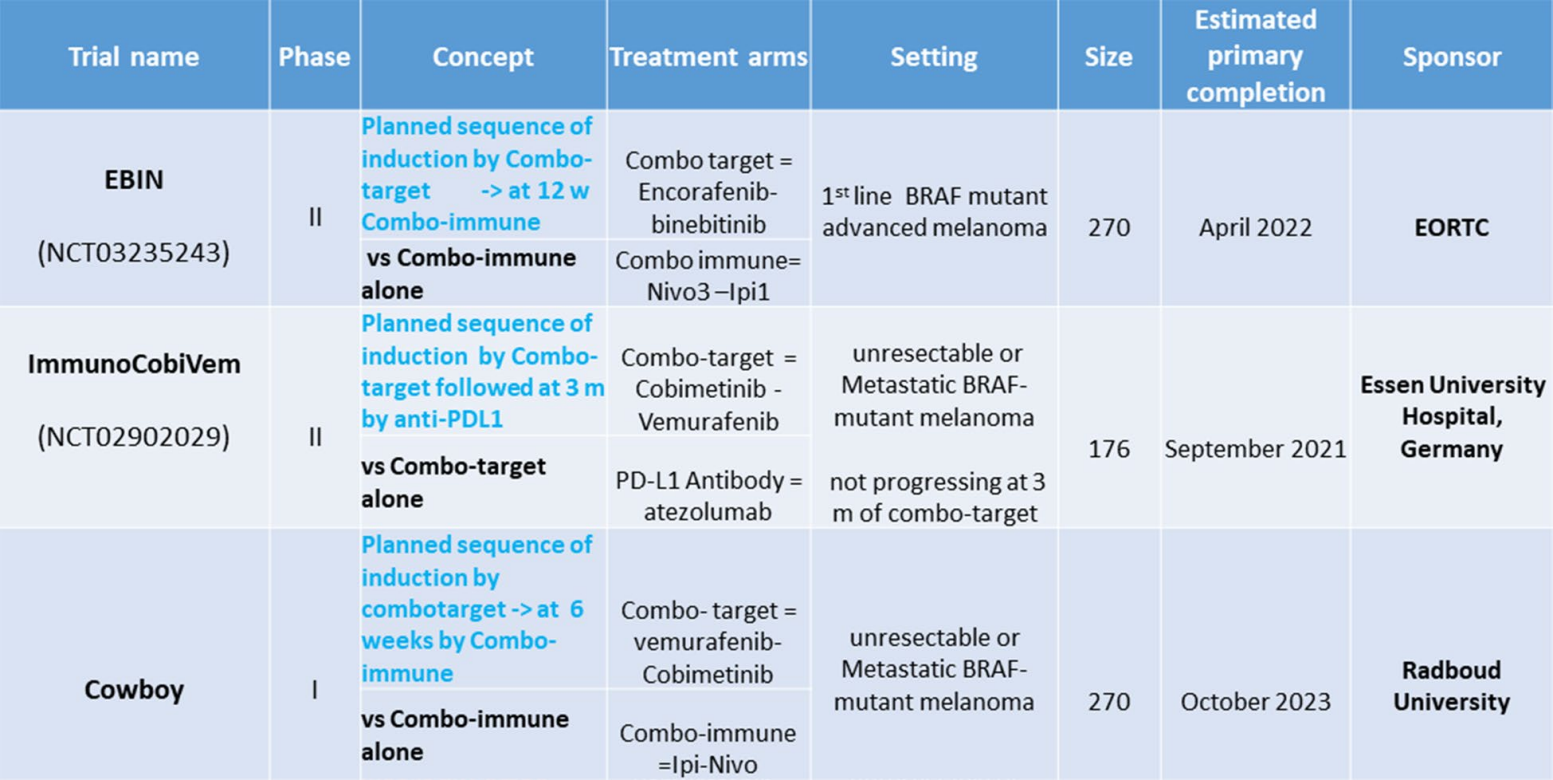
Ongoing trials of BRAF/MEK inhibitor therapy as a starter before immunotherapy

Although discontinuation of checkpoint inhibition may be appropriate in the right situation, the same cannot be said of targeted therapy. Cessation of BRAF/MEK inhibition will result in tumor progression in most cases when the tumor has not been eradicated. In addition, secondary adaptive resistance will develop in most patients at some stage. However, short discontinuation periods are common to manage toxicity and do not seem to reduce the benefit so intermittent treatment may be considered but this requires validation. There may also be a biological rationale for using BRAF/MEK inhibition as an induction treatment before anti-PD-1 immunotherapy but this also requires validation and is being assessed in ongoing trials Fig. 6.

### Omid Hamid: No

The evidence in favour of stopping treatment while on response is weak. Landmark analysis reveals that some patients exhibit sustained durable responses to immune checkpoint inhibitors resulting in prolonged treatment-free intervals. Evidence to support that stopping treatment mitigates potential toxicities is limited and financial burden of treatment should not be the primary concern from a clinician’s perspective.

The CheckMate-153 randomized trial showed a significant PFS benefit in patients with advanced NSCLC who were randomized to continuous nivolumab and were still on treatment at 1-year versus one-year fixed duration nivolumab [HR 0.42, 95% CI 0.25–0.71] [45]. PFS from randomization favored continuous nivolumab for all subgroups. OS also showed a trend that favored continuous nivolumab. In the group that stopped nivolumab at one-year, 43 of 87 (49%) who had a response or SD at randomization had disease progression. Of these, 34 were retreated with nivolumab, meaning some patients were never retreated. Among retreated patients, responses were not that durable and most showed an increase in tumor burden.

Intermittent checkpoint inhibitor therapy may be an option. In patients with metastatic renal cell carcinoma (RCC), those who achieved ≥ 10% reduction in tumor burden on nivolumab entered a treatment-free observation phase with reimaging every 12 weeks [36]. Nivolumab was restarted in patients with a ≥ 10% tumor burden increase and again held when tumor burden reduction was ≥ 10%. Of the 14 patients, five (36%) met the criteria for the intermittent phase and of these one needed to restart therapy and the other four had a sustained response off therapy.

Discontinuation of anti-PD-1 therapy is also a topic in debate in hematological malignancies. In a study of 32 patients with Hodgkin’s lymphoma, 23 discontinued anti-PD-1 treatment after a median duration of 14.6 months due to prolonged response and nine because of unacceptable toxicity; 29 had a CR at time of discontinuation [31]. If discontinuation was an effective strategy, then it seems probable that it would most likely work in Hodgkin’s lymphoma, in which responses are typically deep and durable. However, after a median of 20.8 months from anti-PD-1 discontinuation, more than a third (n = 11) of patients had relapsed/progressed. Of the 29 patients who were in CR at the time of anti-PD-1 discontinuation, estimated 2-year DFS rate was 65% (95% CI 9: 46.6–88.7%). All three patients who were in PR at the time of anti-PD-1 discontinuation had relapsed. Increased risk of relapse at 12 months was based on three main characteristics: the absence of complete metabolic response at the end of anti-PD-1 treatment, prolonged time to achieve best overall response, and older age.

Concerns over increased toxicity with long-term treatment is also probably overstated. In a 5-year analysis of patients with advanced NSCLC treated with pembrolizumab in the KEYNOTE-001 study, incidence of immune-mediated adverse events was similar after three and 5years with little evidence of late-onset or new toxicity [18].

In conclusion, arguments for stopping therapy on response based on cost or toxicity appear to be weak and options for second-line therapy after progression on anti-PD-1 therapy are generally poor. Data from other solid tumors is generally against stopping treatment. It may be an option to individually tailor treatment for selected patients but in general therapy should only be stopped to start another treatment line.

## Key points

**Fig. 7:**
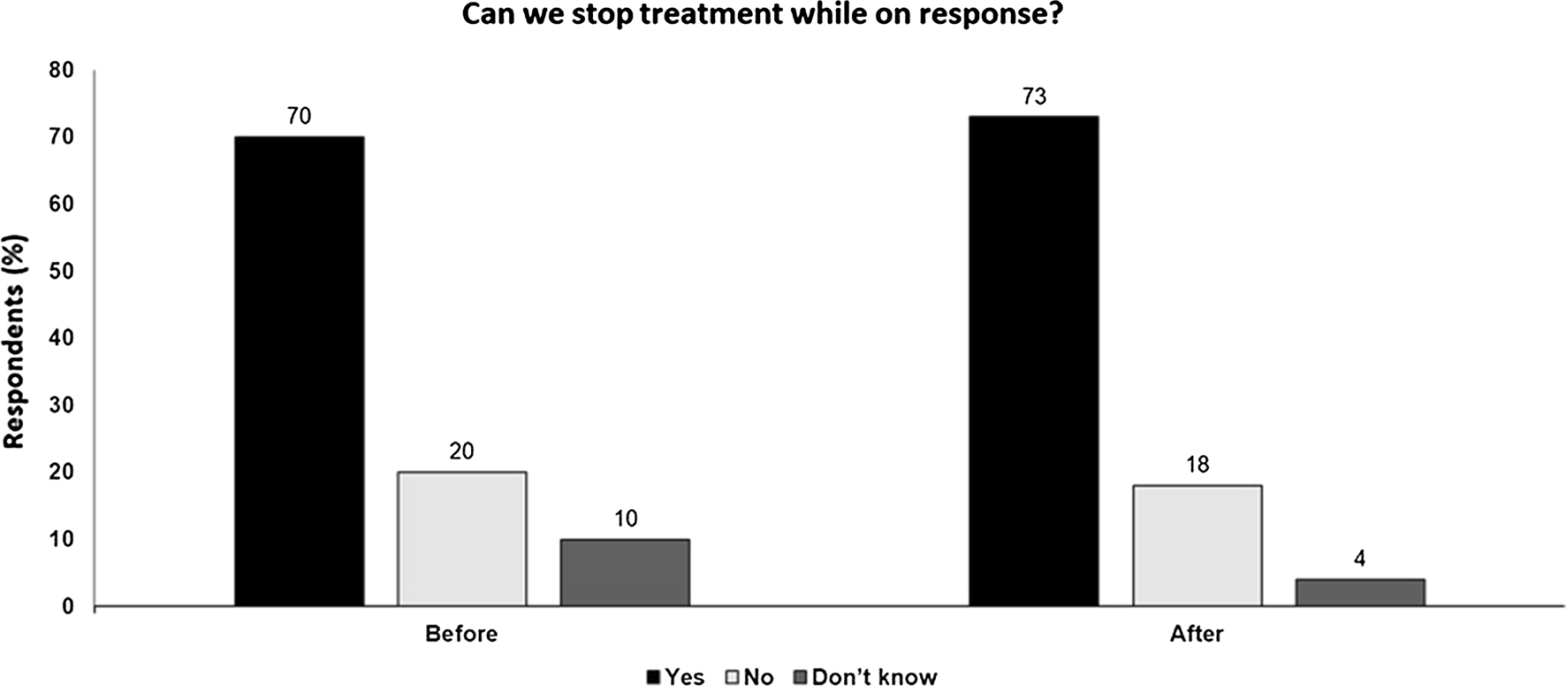
Can we stop treatment while on response? Audience response before and after debate

- Even a short duration of immunotherapy can activate an active immune reaction which may be durable while over-activation via long-term inhibition of PD-1/PD-L1 may be deleterious.
- There are also societal concerns regarding the sustainability, financial cost and burden on the healthcare system of chronic long-term immunotherapy.
- Given this, stopping checkpoint blockade while patients are on response may be possible, although the evidence for such an approach is weak.
- Criteria may be able to help select the best patients for safe discontinuation e.g. intensity of response, but even in the best situation there is always a risk which needs to be explained to the patient.
- Although discontinuation of checkpoint inhibition may be appropriate in the right situation, the same cannot be said of targeted therapy with cessation resulting in tumor progression in most cases when the tumor has not been eradicated (Fig. 7).

## Conclusions

Counterpoint views from leading experts on five controversial clinical issues on Melanoma’s treatment were presented during these Great Debate sessions. Given the constraints of the format and the intended nature of the session, each presentation was not intended as a rigorous assessment of the field but rather provided an opportunity to highlight some important areas of debate. It may be that there are no clear right or wrong answers to these questions; however, it is hoped that these discussions can help focus attention on these issues, stimulating further debate and encouraging the research needed to improve our understanding of different therapeutic approaches and thereby further improve outcomes for patients.

## Data Availability

Not applicable.

## Abbreviations

ACT: Adaptive cell transfer
AJCC: American joint committee on cancer
CAR-T: Chimeric antigen receptor T
CLND: Completion lymph node dissection
CR: Complete response
CT: Computed tomography
CTLA-4: Cytotoxic T-lymphocyte-associated antigen
DFS: Disease free survival
DNA: Deoxyribonucleic acid
EORTC: European Organisation for Research and Treatment of Cancer
FDG-PET: 2-Fluoro-2-deoxy-d-glucose positron emission tomography
HPV16 E7: Human papillomavirus type 16 E7
IL: Interleukin
ILOT: Intralesional oncolytic therapy
MDSCs: Myeloid derived suppressor cells
NSCLC: Non small cell lung cancer
OR: Odds ratio
ORR: Overall response rate
OS: Overall survival
PD-1: Programmed death-1
PD-L1: Programmed death ligand-1
PFS: Progression-free survival
PET: Positron emission tomography
PK: Pharmacokinetic
PR: Partial response
RCC: Renal cell carcinoma
RFS: Recurrence free survival
SD: Stable disease
SNLB: Sentinel lymph node biopsy
TAA: Tumor-associated antigen
TGFβ: Transforming growth factor β
TILs: Tumor-infiltrating lymphocytes
TME: Tumor microenvironment
T-Vec: Talimogene laherperepvec
